# Effects of different light intensity on the growth of tomato seedlings in a plant factory

**DOI:** 10.1371/journal.pone.0294876

**Published:** 2023-11-29

**Authors:** Yifeng Zheng, Jun Zou, Senmao Lin, Chengcui Jin, Mingming Shi, Bobo Yang, Yifan Yang, Dezhi Jin, Rongguang Li, Yuefeng Li, Xing Wen, Shaojun Yang, Xiaotao Ding

**Affiliations:** 1 School of Science, Shanghai Institute of Technology, Fengxian District, Shanghai, 201418, China; 2 Tianchang Fu’an Electronic Co., Ltd., Tianchang, 239300, China; 3 Shanghai Sansi Electronic Engineering Co., Ltd., Shanghai, 201100, China; 4 Shanghai Youyou Agricultural Technology Co., Ltd., Shanghai, 202150, China; 5 Shanghai Academy of Agricultural Sciences, Shanghai, 201403, China; Huazhong Agriculture University, CHINA

## Abstract

Light-emitting diodes (LEDs) were the best artificial light source for plant factories. Red light-emitting diodes (LEDs, R) and blue light-emitting diodes (LEDs, B) were used to obtain different light intensities of uniform spectra, and the greenhouse environment was considered as a comparison. The results showed that root dry weight, shoot dry weight and stem diameter were superior in plant growth under 240 μmolm^-2^s^-1^, additionally, the Dixon Quality Index (DQI) was also best. Under 240 μmolm^-2^s^-1^, the net photosynthesis rate (Pn) was consistent with the greenhouse’s treatment, superior to other experimental groups. The results implied that the PPFD was more suitable for the cultivation of tomato seedlings under the condition of 240 μmolm^-2^s^-1^, and can replace the greenhouse conditions so as to save energy and reduce emissions.

## 1. Introduction

Modern society is currently confronted with a challenging scenario known as a trilemma, where three equally undesirable options exist: (1) a shortage of food, (2) limited resources, and (3) environmental degradation. This trilemma is a global, as well as local and national, issue exacerbated by urban population growth and a declining workforce. To address this trilemma, it is crucial to develop transdisciplinary methodologies based on innovative concepts that significantly enhance food yield and quality while minimizing resource consumption and environmental harm compared to current plant production systems [1,2].

One such system with the potential to achieve this objective is plant factories [3,4] with artificial lighting (PFALs) [5]. PFALs offer several benefits, including high resource use efficiency, increased annual productivity per unit of land, and the production of high-quality plants without the need for pesticides [6,7]. Approximately 1.3 billion tons of food, which accounts for a staggering one-third of the food produced worldwide for human consumption, is wasted annually. Vegetables, in particular, have a high wastage rate. In developing countries, 40% of this loss occurs during post-harvest and processing stages, while in industrialized nations, over 40% is wasted at the retail and consumer levels [8]. Food loss and waste lead to the substantial squandering of resources, including water, land, energy, labor, and capital. Additionally, they contribute to greenhouse gas emissions, further exacerbating global warming and climate change.

Hence, it is crucial to promote local production for local consumption, particularly for fresh vegetables that have high water content. This approach helps in reducing vegetable loss and conserving valuable resources. Fresh vegetables are susceptible to damage during transportation due to their weight. It is well-established that plants with green leaves grow through photosynthesis, which requires essential resources such as water, CO_2_, light energy, and inorganic fertilizers containing 13 nutrient elements. Organic waste materials can be transformed into inorganic fertilizers through decomposition using specific microorganisms. Heat energy released from restaurants, offices, and various industrial facilities at temperatures of 30–60°C can be utilized for greenhouse heating in winter, food and material drying, and other applications. The cultivation of plants for food and various other purposes presents a significant opportunity to reduce resource consumption and waste in urban areas [9].

There are two types of light sources available: natural and artificial. In the practical process of plant factory production [10], which includes both planting and seedling cultivation [11], reliance on natural light sources (such as sunlight) restricts the positioning of carriers like seedbeds or trays. This limitation necessitates a single-layer placement mode to avoid shadowing between plants, resulting in a larger utilization area for the facility. Additionally, considering the resource consumption associated with temperature, humidity, carbon dioxide control, etc., the overall production costs are significantly increased [7]. In comparison, artificial light sources offer several advantages. They can fulfill the light requirements of plants while enabling three-dimensional planting, reducing the utilization area, and lowering production costs. Among the various artificial light sources available, light-emitting diodes (LEDs) have emerged as the primary choice in practical plant factory production. LEDs are highly efficient, consume less energy, have a compact size, and boast a long lifespan.

When tomato seedlings are bred in plant factories, the light intensity, light quality ratio and photoperiod of the artificial light source cannot be controlled in real time, and the tomato seedlings can only be supplemented with fixed light intensity, fixed light quality ratio and fixed photoperiod set in advance. In order to achieve the optimal Dixon Quality Index [12], tomato seedlings need a large light intensity, but greater intensity may inhibit the growth of tomatoes. Therefore, the quality of light intensity has a great influence on the growth and physiological changes of tomato seedlings. When the light intensity is low, the plant is more susceptible to light suppression, resulting in the phenomenon of excessive length and higher plant height. Normally, the net photosynthetic rate (Pn) [11] is consistent with light intensity’s change. There are some plants, such as tomatoes, cucumbers, and strawberries have evolved various mechanisms, including morphological and physiological changes, in order to adapt to various environments. These measures relieve the damage caused by excessive lighting, and ensure the photosynthesis of plants [13]. The percentage absorption of blue or red light by plant leaves is about 90% [14]. Therefore, blue and red light exert a significant influence on plant development and physiology. The combination of red and blue light is increasingly being utilized in both research and commercial horticulture due to their high photosynthetic efficiency at the leaf level, both in the short-term [15] and long-term [16,17]. The absence of either the red or blue light wavelengths [18] leads to photosynthetic inefficiencies. The combination of red and blue light has demonstrated its effectiveness as a lighting source in promoting plant development [19,20] and enhancing plant health [21,22]. The combination of red and blue light in a 7:3 ratio has been found to enhance the fresh weight and dry weight of various plant species, including Lilium, Chrysanthemum, and tomato [23,24]. When cultured under R:B = 7:3 LED light [25,26], the plants exhibited a higher specific leaf area [27,28], which could enhance light absorption. Additionally, the ratio of red to blue light at 7:3 [29,30] resulted in an increase in leaf-level photosynthetic rate (Pn) [24,31].

Previous studies have primarily focused on examining the impact of varying light intensities on plant growth and development in natural sunlight. However, limited information is available regarding the effects of different light intensities on plant growth and development when exposed to a combination of red and blue light. What implications will different artificial light intensities, particularly those involving red and blue light combinations, have on plant growth and development? Furthermore, which artificial light intensity would be optimal for plant cultivation? As a result, it is crucial to investigate the appropriate intensities of red and blue LEDs in combination for industrialized production and to assess the diverse responses arising from low and high light intensities in artificial conditions.

The tomato is a globally distributed crop that is cultivated year-round in China. Large-scale production of young tomato plants predominantly occurs under controlled conditions to meet the growing demand. Within controlled environments, additional lighting is commonly utilized between autumn and spring to foster seedling growth, ensuring consistent high yields and quality throughout the year. Consequently, due to light being the foremost influential element impacting the growth of young tomato plants in controlled environments, further investigation is warranted.

## 2. Materials and methods

### 2.1 Experimental materials

The experiment was conducted in Shanghai from October 2022 to November 2022 in the greenhouse of Youyou Agricultural Technology Co., Ltd. Tomato line Hakumaru was used a plant material, having big fruit size and longer fruiting season [32].

### 2.2 Experimental layout

**Fig 1:**
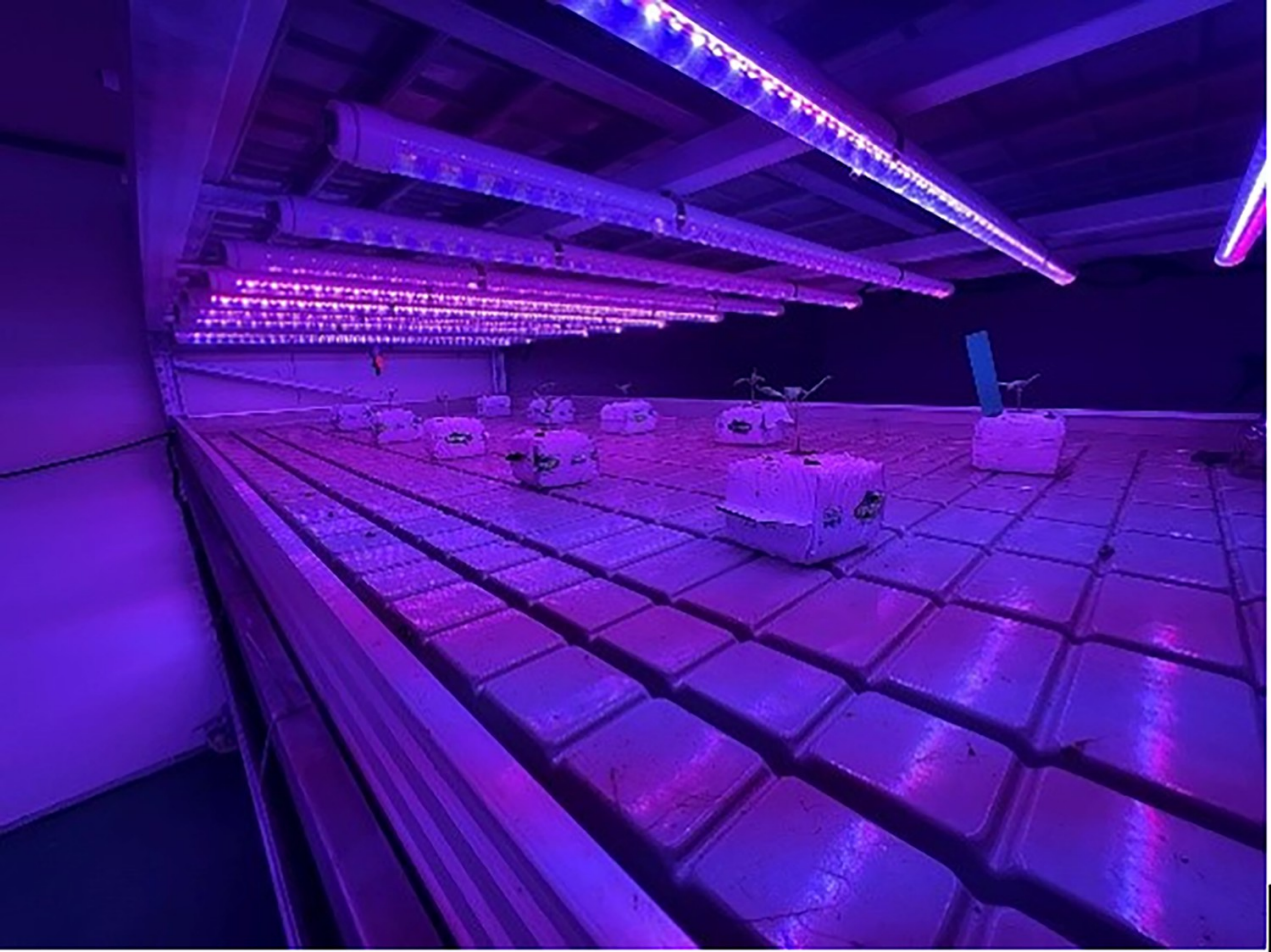
LED fill light equipment.

After sowing by a seeder, tomato seeds (Hakumaru) germinate in an artificial climate box with an indoor air temperature of 22°C and an air humidity of 90% for 72 hours and then sow them in a 240-hole tray, which is placed in an artificial climate box. The photoperiod is stetted by 12h/12h, the temperature is 25°C/20°C, the humidity is 65%~75%, and the LED red-blue ratio is 7:3. When the second leaves were fully expanded, we select plants with consistent growth and transplant them to fill lights (Fig 1). Using grass charcoal: perlite: vermiculite = 3:1:1 (volume ratio) as the cultivation substrate, the tidal nutrient solution irrigation mode was adopted, and the nutrient solution was formulated with 1/2 times the tomato nutrient solution. We use the time control switch (Shanghai Sanshi Technology Development Co., Ltd.) to accurately control the photoperiod (day: night = 12h:12h), control the temperature 25°C/20°C (day/night), and the humidity is 65%~75%. The power supply equipment for the plant supplemental lighting system used in this experiment is provided by Tianchang Fu’an Electronic Co., Ltd. The experiment was repeated by three times, each session lasted 21 days. The experiment used a combination of different LED lamp bead numbers to acquire 4 different light intensity treatments, which were 60 μmolm^-2^s^-1^, 150 μmolm^-2^s^-1^, 240 μmolm^-2^s^-1^, 330 μmolm^-2^s^-1^, using sun light as the comparison, and the red and blue quality ratio of LED was set to a fixed red-blue ratio of 7:3. The four light intensity treatments and sun light are represented by S1, S2, S3, S4, S5, the same below.

### 2.3 Measurement items and methods

#### 2.3.1 Determination of light intensity and spectrum

**Fig 2:**
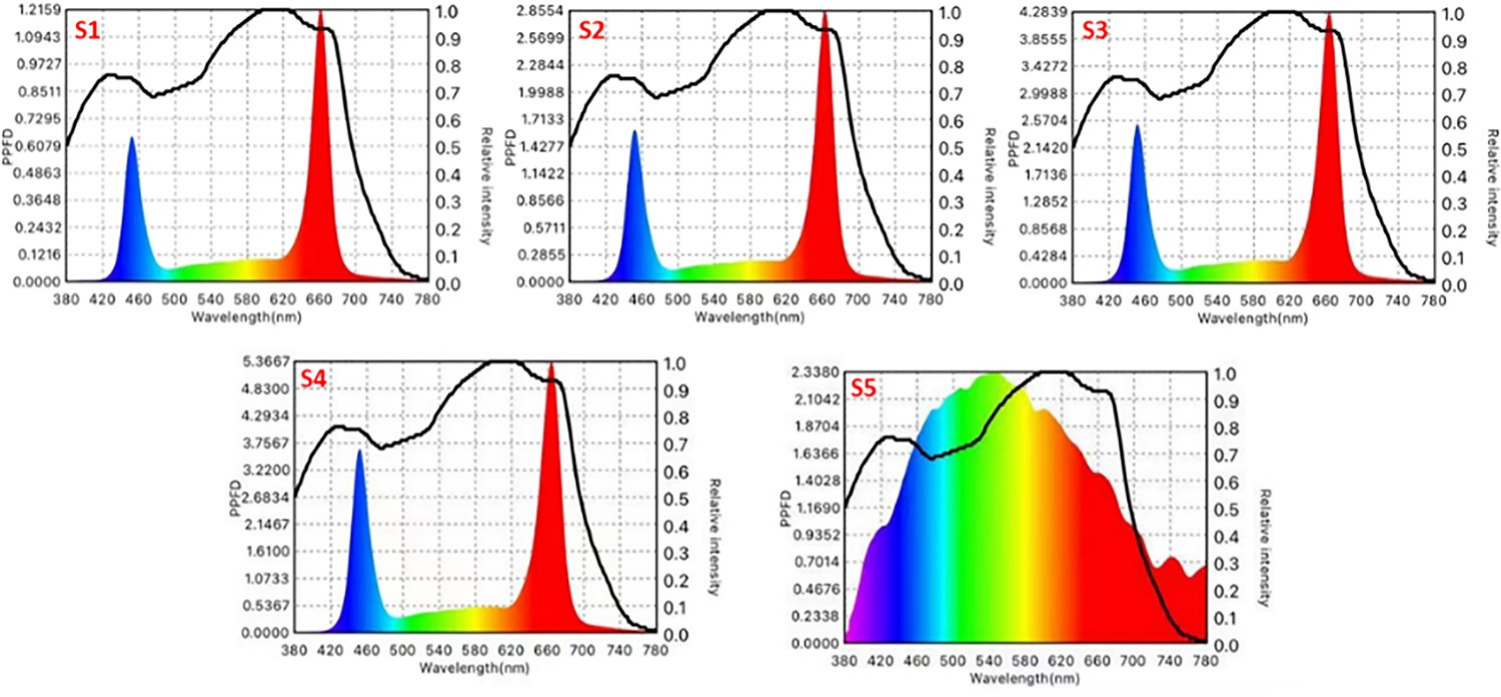
Spectral distribution of S1-S5 under different light intensities.

Prior to the experiment, the spectra of different treatments were determined by spectrometer in a darkroom 20 cm directly below the lamp, as shown in Fig 2 and Table 1, where the black curve is the average of the photosynthesis curve, calculated by McCree [15].

**Table 1:** Major light parameters of treatments.

Treatment	Light intensity (μmolm^-2^s^-1^)	The wavelengths of red light and blue light (nm)
S1	60	662+452
S2	150	662+452
S3	240	662+452
S4	330	662+452
S5	Sun light	Full spectrum

#### 2.3.2 Determination of dry matter accumulation of tomato seedling growth indicators

On the 7th, 14th and 21st days of tomato plants under fill light, 10 plants were randomly selected for each treatment, repeated 3 times, and the plant height of tomato seedlings was measured with a ruler with an accuracy of 0.1cm. Measure the stem diameter above the first segment with an electronic digital caliper, which the accuracy is 0.01 mm; On the 21st day, after rinsing the tomato plant surface substrate and gray layer with distilled water, absorbent paper was used to absorb the moisture on the plant surface, and then the fresh quality of tomato seedling leaves, stems and roots was measured by an electronic balance, which the accuracy is 0.01g. After the tomato plants were fixed at 105°C for 15min, dried in an electric blower drying oven at 80°C for 48h to a constant amount. The dry mass of each part was weighed with an electronic balance, which the accuracy is 0.01g. At last, the DQI [12] was calculated as followed:

$$Quality\;index=\left(\frac{stem\;thickness}{plant\;height}+\frac{root\;dry\;weight}{shoot\;dry\;weight}\right)\times total\;dry\;weight$$

#### 2.3.3 Determination of chlorophyll content

Treatments were made after the 21st day, and tomato leaves were measured by using a chlorophyll analyzer [11] from Kinkolida. Each control group selected 10 tomato leaves. The accuracy of the chlorophyll analyzer was 0.1.

#### 2.3.4 Determination of photosynthetic properties

Tomato seedlings are treated at different light intensities after 21 days, the net photosynthetic rate (Pn), stomatal conductance (Gs), and intercellular CO_2_ concentration (Ci) of tomato seedlings were measured by CIRAS-3 portable photosynthesis/fluorescence instrument of Lufthansa under different light intensity treatment. Five plants were selected for each treatment, and three leaves were selected for each plant for assay. The gas exchange method is described below.

The infrared radiation is absorbed by gas molecules when passing through CO_2_ gas (or water vapor), resulting in a decrease in transmitted infrared energy. The amount of absorbed infrared energy is related to the absorption coefficient (K) of the gas, gas concentration (C), and the thickness of the gas layer (L), following the Lambert-Beer law, which can be expressed by the following equation:

$$E=E_{0}e^{KCL}$$

E_0_ represents the energy of incident infrared light, while E represents the energy transmitted through the infrared light. According to the above formula, the concentration of CO_2_ or water vapor in the measured gas can be determined. Infrared gas analyzers can only measure the concentrations of CO_2_ and water vapor. To measure photosynthetic rate, it must be combined with the gas pathway system. Connect the infrared analyzer with the assimilation chamber to form an open gas circuit system, providing a stable CO_2_ gas source (AIR) to the assimilation chamber. Insert the leaf into the assimilation chamber (C) and provide appropriate illumination (PAR). After the CO_2_ difference (CO_2_d) and water vapor difference (H_2_Od) between the reference and analysis chambers stabilize, record these two differences. Accurately measure the flow rate (F) of the assimilation chamber, and then calculate the photosynthetic rate (Pn) and transpiration rate (E) based on the leaf area (S).

#### 2.3.5 Statistical analysis

SPSS 22.0 was used for ANOVA and significance analysis (P < 0.05). The experimental results underwent analysis of variance (ANOVA) followed by the Tukey test. Origin Pro 2022 was used for chart production.

## 3. Results

### 3.1 Effects of different light intensity treatments on tomato plant growth

**Fig 3:**
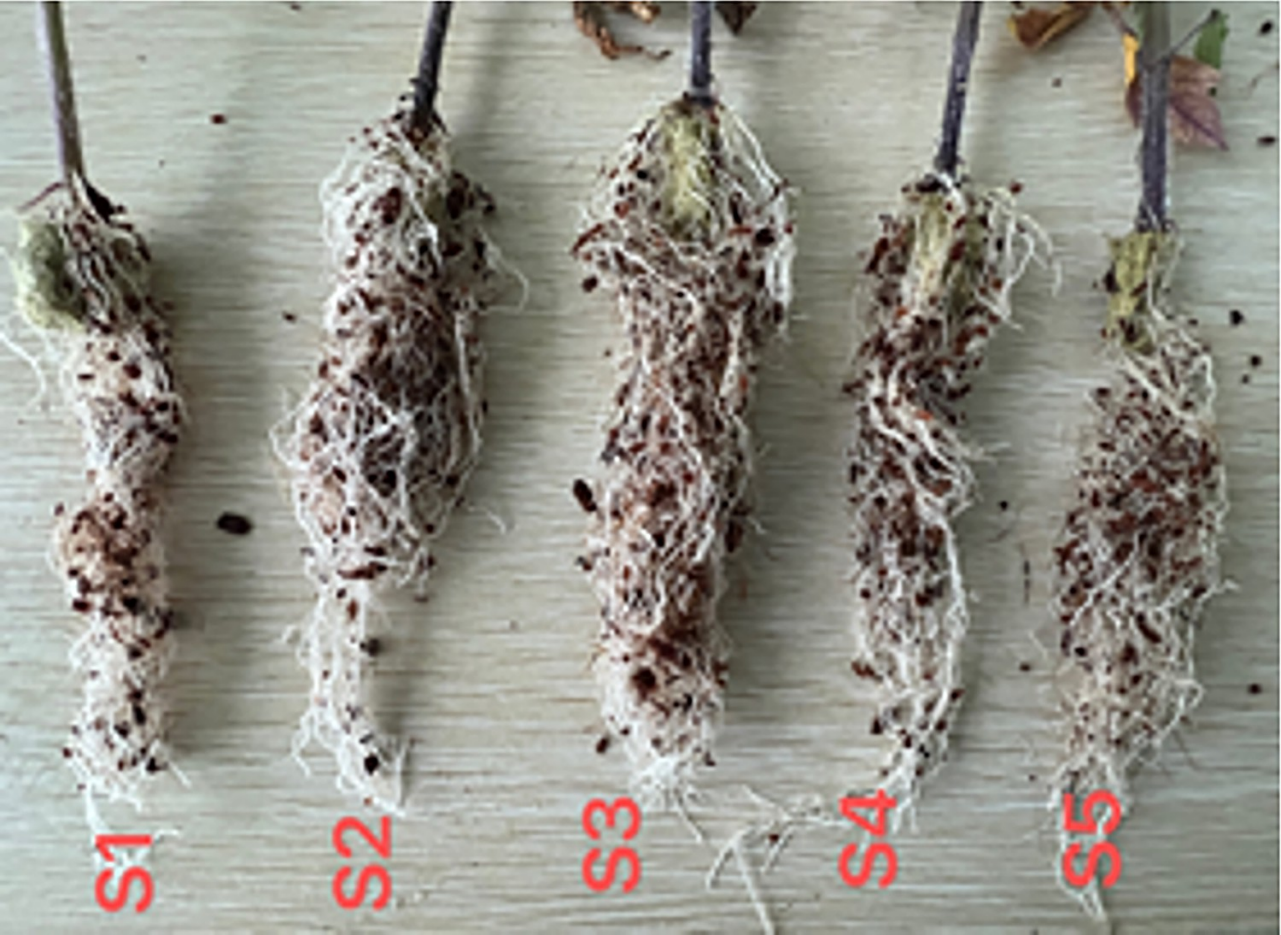
Root system comparison chart of tomato seedlings.

Experiments demonstrated significant variations in the morphology of tomato seedlings under different light intensities (Table 2 and Fig 3). The results predicted that dry weight and other parameters of tomato seedlings were the lowest in S1 treatment. Compared with other treated seedlings, the stem diameter was relatively largest under PPFD irradiation of 240 μmolm^-2^s^-1^, but the optimal state doesn’t increase as PPFD increases. It is clear that PPFD of 330 μmolm^-2^s^-1^, the stem diameter is not as large as 240 μmolm^-2^s^-1^, indicating that it is important to find out the light intensity suitable for the growth of tomato seedlings. At the same time, it can be seen that the light intensity is weaker, the plant height of tomato seedlings is higher, indicating that at 60 μmolm^-2^s^-1^, because of insufficient light, the plant had the phenomenon of apprenticeship, which is not conducive to the growth of tomatoes. From the significant difference in the strength index, it can be seen that tomato seedlings were the strongest under the treatment of S3, followed by S5, which is significantly compared with other treatments.

**Table 2:** Effects of different light intensities on morphology of young tomato plants. Different letters in columns indicate statistically significant differences (P < 0.05).

Light treatment	Root dry weight (g)	Shoot dry weight (g)	Total dry weight (g)	Plant height (cm)	Stem diameter (mm)	Dixon quality index
S1	0.99±0.05d	0.42±0.03d	1.41±0.08d	16.60±0.99a	3.57±0.18b	3.35±0.17cd
S2	1.24±0.03b	0.75±0.02a	1.99±0.06b	13.65±0.98bc	4.10±0.14a	3.34±0.08d
S3	1.63±0.08a	0.67±0.07b	2.30±0.15a	12.84±0.55cd	4.23±0.16a	5.72±0.18a
S4	1.17±0.01bc	0.58±0.01c	1.75±0.02c	11.70±0.79d	3.75±0.18b	3.59±0.05c
S5	1.14±0.01c	0.32±0.02e	1.46±0.03d	14.51±1.04b	3.25±0.02c	5.20±0.26b

### 3.2 Effects of different light intensity treatments on chloro-phyll in leaves of tomato plants

**Fig 4:**
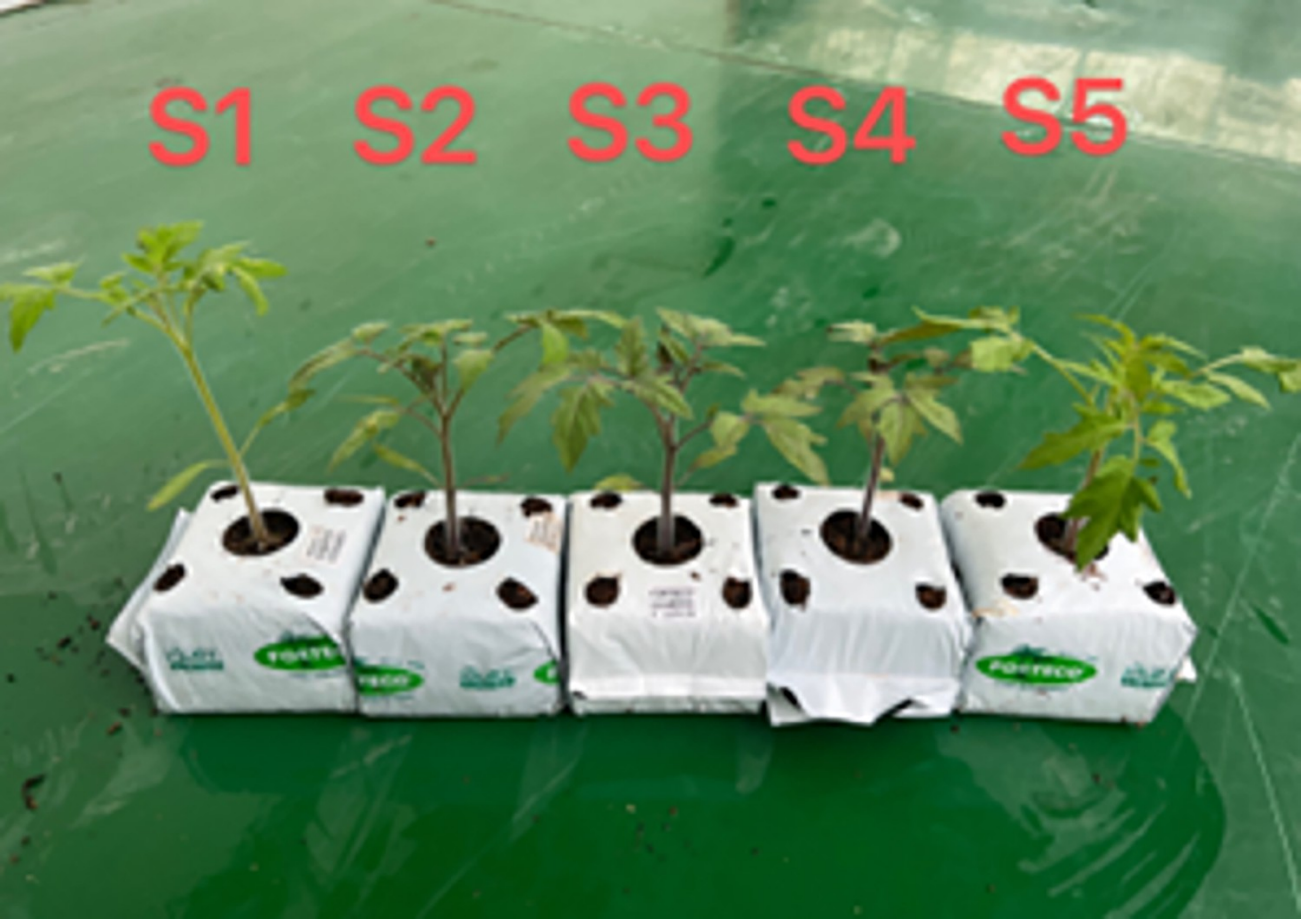
Tomato seedling growth comparison chart.

**Fig 5:**
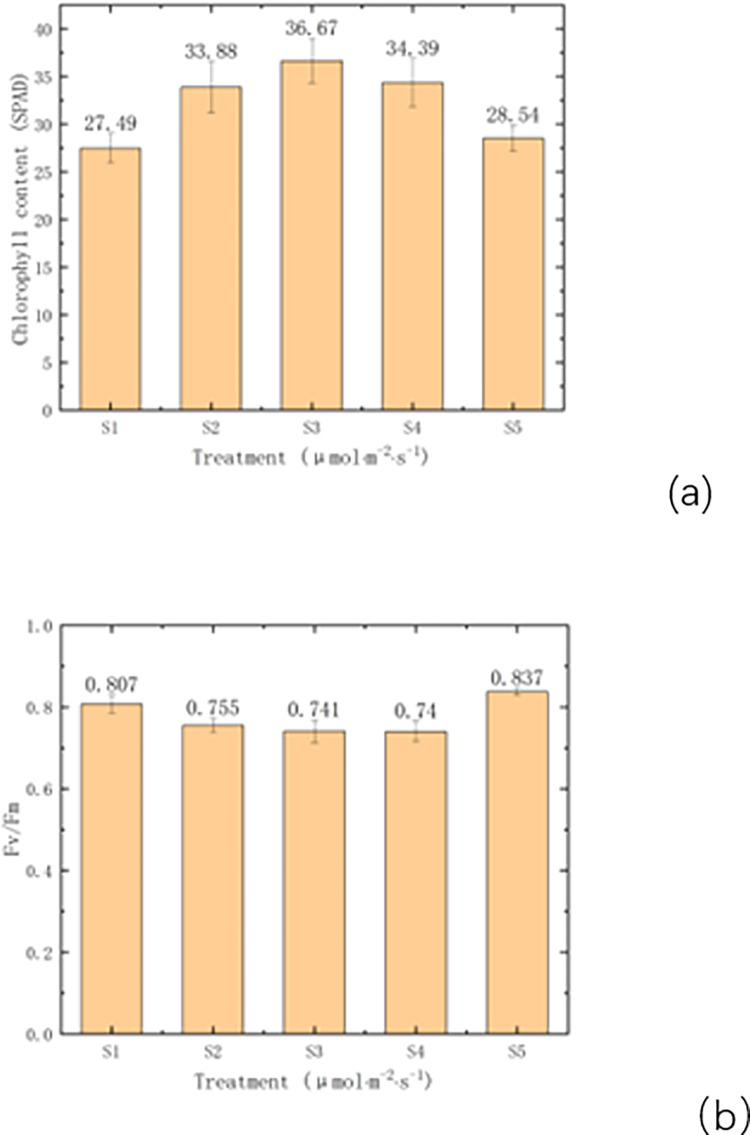
(a). Effects of different light intensity on chlorophyll content of tomato seedlings. (b).Effects of different light intensity on Fv/Fm content of tomato seedlings.

The amount of photosynthetic pigment absorption is first of all related to light quality. In this experiment, we control the optimal light-to-quality ratio (R:B = 7:3) [32], according to Fig 4, we can intuitively see the gap from the leaf color, the leaves of S1 and S5 are lighter in color, and the tomato’s leaves in the middle three groups are darker; From Fig 5(A), it is more fully evident that under the irradiation of PPFD (photosynthetic photon flux density) of 240 μmolm^-2^s^-1^, the chlorophyll content of tomato seedling leaves is the highest, reaching a value of 36.67. Furthermore, the chlorophyll content does not continue to increase with the increase of PPFD. Under the condition of S5, the chlorophyll content of tomato leaves did not accumulate sufficiently, indicating that the LED light source used for artificial light environment control has a positive effect on promoting chlorophyll content in tomato leaves. From Fig 5(B), We can observe that PSII in groups S1 to S4 is lower than that of S5. The reason for the lower PSII in the first four groups is the insufficient water availability in the artificial light environment during the experiment, which resulted in the malfunction of the moisture regulation system.

### 3.3 Effects of different light intensity treatments on photo-synthetic characteristics of tomato plants

**Fig 6:**
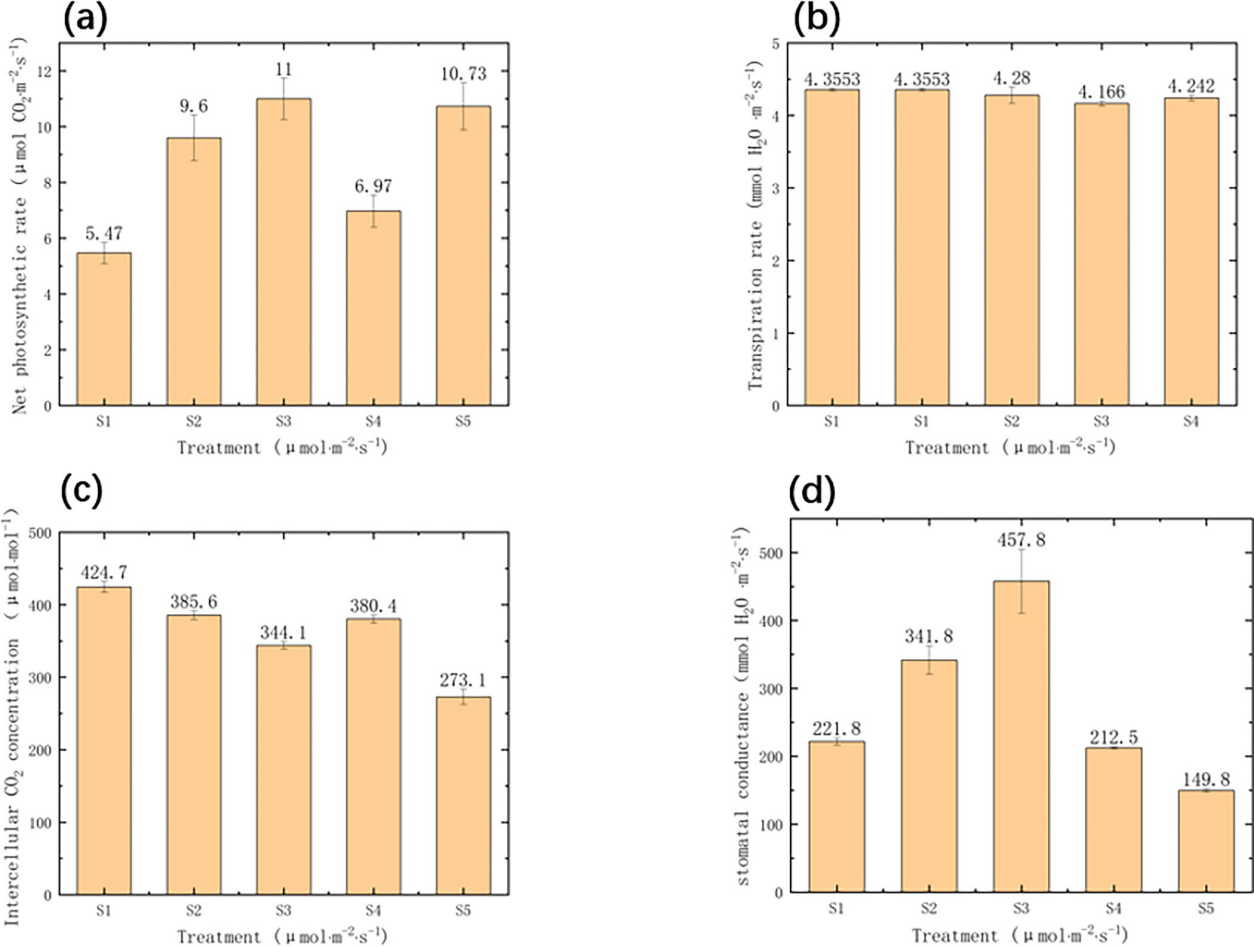
(a) Effects of different light intensities on net photosynthetic rate (Pn) tomato seedlings. (b) Effects of different light intensity on transpiration rate of tomato seedlings. (c) Effects of different light intensity on intercellular carbon dioxide concentration in tomato seedlings. (d) Effect of different light intensity on stomatal conductance of tomato seedlings.

From Fig 6(A), it can be seen that the net photosynthetic rate is the highest at 240 μmolm^-2^s^-1^, followed by greenhouse natural light conditions. Similarly, as the PPFD increased from 60 μmolm^-2^s^-1^ to 240 μmolm^-2^s^-1^, the net photosynthetic rate also increased, but when it reached 330 μmolm^-2^s^-1^, the net photosynthetic rate of tomato seedlings decreased instead. Based on Fig 6(B), it can be concluded that there was not a significant difference in transpiration rate among the four artificial light environment treatments. However, compared with the greenhouse natural light seedlings, the transpiration rate was significantly higher in the artificial light environment treatments. There is a certain correlation between stomatal conductance and intercellular CO_2_ concentration in Fig 6(C) and 6(D). It can be seen that among the four groups with different PPFD levels in the artificial light environment control, the stomatal conductance was the highest and the intercellular CO_2_ concentration was the lowest under the treatment of 240 μmolm^-2^s^-1^. This indicates that more CO_2_ is being used for photosynthesis and simultaneously demonstrates that the net photosynthetic rate of the tomato seedlings is optimal under this condition.

## 4. Discussion

The light source plays an important role in the growth and development of tomatoes, providing energy for tomatoes on the one hand, and regulating tomato plant morphology on the other hand [33,34]. The architecture of plants is partially regulated by light signals received from the environment [24]. Light serves as the energy source for photosynthetic organisms, and the intensity of light plays a crucial role in plant growth. Under low light conditions, plant growth and productivity are hindered due to the impact on gas exchange. Conversely, excessive light intensity can have detrimental effects on the photosynthetic apparatus. A large amount of research has shown that the growth and development of tomato, leaf morphology, and accumulation of dry matter are significantly affected by light intensity [18,35–37]. This study found that the DQI of tomatoes is at its maximum under the condition of 240 μmolm^-2^s^-1^. The DQI does not gradually increase with the increase of PPFD. This is similar to the research of Fan, X.-X. [11] on tomatoes. However, this study is not exactly the same as his light saturation point, indicating that different tomato varieties have different response mechanisms to light intensity, which is consistent with the research of Wang, L. W. [38]. From the root comparison diagram in Fig 4 above, it can be found that the degree of root development also reaches its optimum at 240 μmolm^-2^s^-1^, indicating that under this treatment, the rate of accumulation of dry matter and equality in tomato plants accelerates, further verifying the DQI. This is consistent with the research of Shimizu, H. [39] and others on lettuce in plant factories.

For the chlorophyll content of tomato leaves [39], SPAD [40] reflects the degree of leaf greenness, and then the actual nitrate content of tomatoes can be understood. Therefore, this is how to judge whether the nitrogen content in coconut coir blocks meets the growth requirements of tomato plants. In this experiment, the treatment with the highest chlorophyll content was 240 μmolm^-2^s^-1^. The chlorophyll content of 150 μmolm^-2^s^-1^ and 330 μmolm^-2^s^-1^ did not differ significantly from it, but they were much higher than 60 μmolm^-2^s^-1^ and natural light (W). This indicates that the PPFD always affects the chlorophyll content of tomato leaves, which is similar to the analysis and verification of Jiang, C. [41,42]. If the absorbed excessive light energy by the photosynthetic apparatus cannot be dissipated quickly, it can decrease the efficiency of photosynthesis and lead to photoinhibition and potential damage to the photosynthetic reaction center. For example, high light stress can easily cause photoinhibition in photosystem I, and it also inhibits the repair of photosystem II. [42]. In Fig 6 of this experiment, as the PPFD increased, the energy capture efficiency of the photosystem II reaction center (Fv/Fm) actually decreased. This indicates that although high PPFD increases parameters such as the dry matter and DQI of tomatoes, it also causes some damage to the cells of tomato leaves, which is consistent with the research of Zsiros, O. [43].

Stomata play a vital role in facilitating the exchange of water and air with the external environment. The conductance of stomata is influenced by light intensity, which enhances the proton motive force [44,45]. Additionally, the development of stomata seems to be associated with light intensity [31]. As shown in Fig 6(C) and 6(D), with the increase of PPFD, the stomatal conductance also increases, reaching its maximum at 240 μmolm^-2^s^-1^. This indicates that the opening of the stomata in tomato leaves is the largest at this point. On the other hand, the concentration of intercellular carbon dioxide is also the lowest at this point. Therefore, the exchange efficiency between tomato leaves under this treatment and the external environment is optimal, resulting in higher photosynthetic efficiency. This is consistent with the research of Gorton, H. L. [46]. As shown in Fig 6(B), after the four treatments with artificial light environment control, the transpiration rate of tomato leaves was significantly higher than that of natural light (W) in the greenhouse, which is consistent with the analysis results of Jolliet, O. [47]. It is clear in Fig 6(A) that the net photosynthetic rate of tomato plants is optimal at 240 μmolm^-2^s^-1^, and slightly higher than that in greenhouses. The current study revealed that a photosynthetic photon flux density (PPFD) of 240 μmolm^-2^s^-1^ resulted in the highest net photosynthesis (Pn). Based on these findings, we observed a similar trend between plant height, stem diameter, stomatal frequency, and Pn activity. Additionally, a higher stomatal frequency can facilitate CO_2_ uptake, thereby sustaining a higher level of photosynthetic activity. Therefore, we speculate that the increase in Pn associated with a PPFD of 240 μmolm^-2^s^-1^ is likely influenced by stem diameter, higher stomatal frequency, and the Dixon Quality Index.

## 5. Conclusions

The results clearly demonstrate that, compared to other light treatments, from 60 to 330 μmolm^-2^s^-1^, the biomass and heath index of young plants were better. More important, 240 μmolm^-2^s^-1^induced the highest energy efficiency and activity of Pn. In the research, we found that there was no substantial gain from a PPFD above 240 μmolm^-2^s^-1^. Therefore, this experiment verified a conclusion that the treatment of tomato seedlings by LED light source under the artificial light can replace the natural light in the greenhouse. Compared with the greenhouse, this method used for tomato seedling breeding in the plant factory with artificial lighting will greatly reduce the cost and improve the energy efficiency.
